# Imaging and Patient Selection for Transcatheter Tricuspid Valve Interventions

**DOI:** 10.3389/fcvm.2020.00060

**Published:** 2020-05-05

**Authors:** Mirjam G. Winkel, Nicolas Brugger, Omar K. Khalique, Christoph Gräni, Adrian Huber, Thomas Pilgrim, Michael Billinger, Stephan Windecker, Rebecca T. Hahn, Fabien Praz

**Affiliations:** ^1^Department of Cardiology, Inselspital, University Hospital Bern, Bern, Switzerland; ^2^Columbia University Medical Center/NY Presbyterian Hospital, New York, NY, United States

**Keywords:** tricuspid regurgitation, valvular heart disease, tricuspid interventions, imaging of tricuspid valve, annuloplasty, edge-to-edge repair, caval stent, valve replacement

## Abstract

With the emergence of transcatheter solutions for the treatment of tricuspid regurgitation (TR) increased attention has been directed to the once neglected tricuspid valve (TV) complex. Recent studies have highlighted new aspects of valve anatomy and TR etiology. The assessment of valve morphology along with quantification of regurgitation severity and RV function pose several challenges to cardiac imagers guiding transcatheter valve procedures. This review article aims to give an overview over the role of modern imaging modalities during assessment and treatment of the TV.

## Introduction

Since the recent emergence of percutaneous valve interventions as a possible alternative to surgery or medical treatment, tricuspid valve (TV) disease has attracted growing attention. Although tricuspid regurgitation (TR) of any severity is present in about 70% of the population (1), in the past this entity has been neglected in daily clinical practice. Severe TR affects around 4% of the population over 75 years of age, with higher prevalence in women, elderly patients, and in those who already underwent open-heart surgery for left-sided heart valve disease. This number is expected to rise in the future due to population aging (2–4).

Multiple observational studies have reported worse survival in patients with severe TR, irrespective of left and right ventricular function, pulmonary artery pressure, age, gender and co-morbidities (2, 5–10). In patients undergoing left-sided heart valve surgery or interventional treatment, the presence of relevant TR has been identified as a predictor of poor outcomes (11, 12). A recent propensity-matched cohort study showed that transcatheter TV interventions might be able to improve prognosis compared to medical treatment alone (13).

Imaging the TV and grading TR is challenging as transfer of existing knowledge and recommendations from the left side of the heart is not always possible. In contrast to the mitral valve, the TV operates in a low pressure environment with slower jet velocity. In addition, valve geometry, TR proximal flow convergence zone, and jet morphologies are more complex, making the usual tools and geometrical assumptions less accurate (14). The high variability of TR depending on small preload changes (e.g., during the respiratory cycle) (15), represents an additional difficulty (2, 16).

## Morphology and Anatomical Relations

Autoptic studies have enhanced the anatomical understanding of the TV (17–19). Indeed, despite its name, the TV is truly tricuspid in only 57% of the investigated subjects (20). In the remaining 43%, it is quadricuspid with an additional leaflet, generally located between the septal and the posterior ones.

The healthy tricuspid annulus has a three-dimensional saddle-shaped elliptical geometry (15, 21–23). Its anterior and posterior portions are muscular, whereas the septal part is more fibrous, which explains predominant antero-posterior annular dilation as well as the spherical and planar shape of the annulus in patients with severe functional TR (11, 24). The tricuspid annulus is contiguous to several important anatomical structures (25). The postero-septal portion is close to the ostium of the coronary sinus, delimiting the triangle of Koch, where both the atrio-ventricular (AV) node and His-bundle are located. The antero-septal aspect of the annulus is situated next to the right ventricular outflow tract and the right coronary artery ostium. In its further course, the right coronary artery circumscribes the anterior and posterior portion of the annulus (11, 26) which exposes it to a risk of compression, kinking or occlusion during annuloplasty procedures, especially when located close to the hinge point of the TV leaflets. Although no data exist, a distance of <2 mm has been suggested as a possible cut-off and is found in 13–28% of the patients (27, 28).

## Etiology and Mechanisms of TR

TR etiology can be divided in primary (or organic) TR due to leaflet abnormalities, and secondary (or functional) TR due to annular and right atrial, or right ventricular dilation (25). Diseases leading to leaflet deformation can be either acquired, such as rheumatic or carcinoid heart disease, endocarditis, trauma, or congenital, like Ebstein's anomaly and endocardial cushion defect (29). Functional TR accounts for up to 94% of moderate to severe TR cases, with 49% occurring in the context of left-sided valvular disease, 23% concomitantly to relevant pulmonary hypertension (systolic pressure ≥50 mmHg), 13% in association with left ventricular dysfunction and 8% in isolation without any of the previously mentioned causes (2). Isolated TR was an independent predictor of all-cause mortality even after adjustment for various confounders (2). Increasing TR severity correlates with a higher cardiovascular mortality rate (2, 5, 9, 10).

TR leads to volume overload and further RV and RA dilation, resulting in annulus dilation, papillary muscle displacement and leaflet tethering, also influenced by elevated pulmonary artery pressure, further aggravating valve dysfunction (30, 31). TR not only has a mechanical effect on the right heart structures, but also increases stiffness of the RA, possibly due to chronic inflammatory processes and formation of interstitial fibrosis (32). Patients with associated right ventricular dysfunction, independently from RV dilation, have a particularly unfavorable clinical prognosis (33).

Chronic atrial fibrillation can be either the cause or the result of TR. Studies report a high overall prevalence of chronic atrial fibrillation in patients with moderate or severe TR (up to 68%) with a yearly incidence of 28% in the setting of associated left-sided valvular heart disease and 13% in isolated TR (2). Conversion to sinus rhythm may effectively reduce TR (34).

TR in the presence of cardiac implantable electronic devices-leads (CIED) is a topic of growing concern due to the rising number of implantations. New-onset significant TR after CIED placement has been observed in up to 38% of the patients, either resulting from direct valve injury or adverse interaction with the leaflets, most commonly affecting the septal leaflet (35), or the subvalvular apparatus (36, 37). Due to frequently associated left ventricular dysfunction and comorbidities acting as confounders, the causality of the higher mortality observed in patients with CIED-related TR is difficult to establish (38). The localization of the lead appears to influences the severity of TR. While a lead implanted in the interventricular septum has a higher risk of leaflet impingement, a more commissural or central position seems less problematic (37, 39). Interestingly, leadless pacemaker may also contribute to TV dysfunction because of either ventricular dyssynchrony induced by RV pacing or unintended interaction with the subvalvular apparatus (40, 41).

## Grading Tricuspid Regurgitation Severity

Imaging the TV is associated with particular challenges summarized in Table 1. TR severity should be assessed in an integrative way using various echocardiographic parameters, as well as adjunctive imaging modalities such as multislice computed tomography (MSCT) and cardiac magnetic resonance imaging (CMR), when echocardiographic quality is poor or severity parameters are discordant (14).

**Table 1:** Challenges of imaging the tricuspid valve.

**Challenges of TV Imaging**
• Variable and fragile anatomy
• High (pre- and after-) load dependancy
• Low pressure environment / slower jet velocity
• Presence of CIED-leads
• Artifacts from left-sided bioprosthetic valves
• Limited evidence and experience
• Not or insufficiently validated cut-off values

Due to the anterior position of the RV close to the chest, transthoracic echocardiography usually provides satisfactory imaging quality for severity grading (42). Advanced anatomical assessment typically requires a dedicated 3D transesophageal echocardiography (TEE) study owing to higher spatial resolution.

An integrative approach considering identical parameters using different imaging modalities is likely to improve the diagnostic accuracy.

Quantitative and semi-quantitative parameters considered useful for grading TR include the following:

### Color Jet Area

Echocardiographic measurement of the color jet area using the 4-chamber, RV inflow or subcostal views is indicative of severe TR if the jet area exceeds 10 cm^2^. It is physiologically influenced by direction, momentum and velocity of the jet and the systolic pressure difference between RV and RA (43), and technically by the color scale and wall filter settings, as well as the transducer frequency (14). Importantly, in very severe TR an early equalization of the pressure between the RV and the RA can occur, leading to a very low velocity with almost no visible jet (30).

### Flow Reversal in the Hepatic Veins

Flow reversal into the hepatic veins is a specific parameter (if present: >85% probability of severe TR) (44), but with rather low sensitivity, as the venous flow patterns depend on various factors including RA dimensions and compliance, RV function, as well as atrial fibrillation or pacemaker stimulation (11, 30). A reflux of contrast medium into the inferior vena cava with enhancement of the mid to distal hepatic veins on MSCT is also considered highly specific for significant TR (45).

### Tricuspid Inflow Velocity

Tricuspid inflow velocity can be used as a complementary method to grade TR. A peak tricuspid E-wave velocity >1.0 m/sec has been associated with right ventricular pathology (46) and severe TR (47). The tricuspid inflow velocity represents a simply obtained measurement, but has to be carefully interpreted in the context of age and heart rate.

### Vena Contracta (VC)

**Figure 1:**
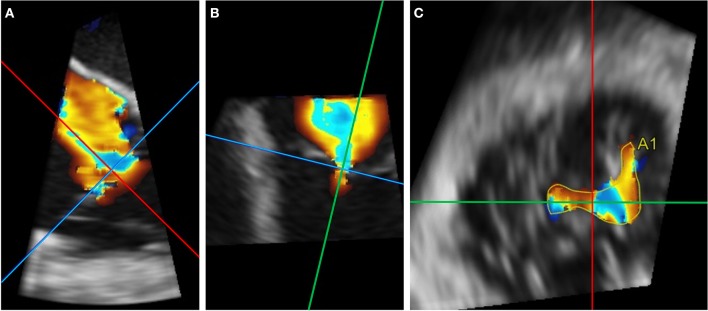
Example of 3D Doppler VC area using multiplanar reconstruction. **(A,B)** Reformation planes are aligned at the height of the 2D vena contracta during systole in two different planes. **(C)** A1 measures 1.65 cm^2^ in this case.

VC is defined as the narrowest width of the color regurgitant jet and is usually measured directly below the proximal flow convergence zone. Severe TR is defined as a VC width ≥ 7 mm in the RV inflow view according to current guidelines, a value that has been associated with worse cardiovascular outcomes (1, 48–50). Due to triangular and elongated shape of the regurgitant orifice in TR, a single 2D measurement of the VC only insufficiently reflects the anatomical reality. Song et al. (51) have proposed the use of different VC width cutoff values for severe TR depending on the plane of the view: 8.4 mm in the septo-lateral and 12.6 mm in the antero-posterior view, respectively. Dahou et al. (52) have also suggested measurement in two orthogonal planes with an average VC cutoff of ≥ 9 mm. The VC widths measured in the septo-lateral projection were 3.9 ± 3.7 mm smaller than the one measured in the antero-posterior direction and discrepancies were found to worsen with increasing TR severity. To overcome these limitations, measurement of the 3D Doppler VC area using multiplanar reconstruction (Figure 1) may be considered (52, 53) Cut-offs for severe TR ranging from 0.37 to 0.75 cm^2^ have been proposed (51–53).

### Regurgitant Volume

The regurgitant volume can be derived from the stroke volumes (SV) assessed by quantitative Doppler and is calculated as the antegrade tricuspid diastolic SV minus the left ventricular or right ventricular outflow forward SV. In the presence of more than mild aortic regurgitation, the right ventricular forward SV should be preferred, and vice versa. In both cases, the SV is obtained from the diameter (D) and the velocity time integral (VTI) of either the right or left ventricular outflow tract as (D/2)^2^ × π × VTI.

The tricuspid diastolic SV by quantitative Doppler is calculated through multiplication of the tricuspid annular area (preferably measured on 3D multiplanar reconstruction) by the pulsed-wave Doppler VTI through the annulus (54, 55). The tricuspid diastolic SV may be overestimated in case of heterogeneous and complex annular flow patterns (54). Despite interobserver variability, the volume derived from quantitative Doppler assessment correlates well with other echocardiographic parameters (56) and has a prognostic value in patients with TR and reduced left ventricular function (8).

Using 4D MSCT the regurgitant volume and fraction are derived from the difference between RV and LV stroke volume obtained by ventricular volumetry. Higher saptial resolution may represent an advantage, but cutoffs to grade TR severity have not been established yet (14, 57). Table 2 shows an example of a CT protocol dedicated to the tricuspid valve. The use of a mixture of saline/contrast is considered helpful to increase the contrast travel time and minimize streak artifacts (58).

**Table 2:** Example of a dedicated computed tomography protocol for the tricuspid valve.

**Examination protocol for ECG-gated computed tomography of the TV**
• 2 × 128 row stellar detector (e.g., Siemens SOMATOM Definition Flash)
• Inspiratory breath-hold, single-volume acquisition
• Retrospective ecg-triggered acquisition over the whole cardiac cycle (0–100% R-R interval)
• Isotrophic resolution 0.33 × 0.33 mm, crossplane 0.30 mm; gantry rotation time 280 ms; temporal resolution 750 ms
• Tube voltage 100–120 kV, tube current 240 reference mAs (care dose)
• Intravenous injection of non-ionic contrast agent (iopromide) • 50 ml contrast medium at a rate of 4 ml/s, followed by • 30 ml contrast medium at a rate of 3 ml/s, followed by • 20 ml of saline at a rate of 4 ml/s • total contrast volume = 80 ml
• Real-time bolus tracking with automated peak enhancement detection with region of interest ascending aorta, based on a threshold of 120 Hounsfield units
• Reconstruction of the 3D data set from the contrast-enhanced scan at 5% increments throughout the cardiac cycle with a slice thickness of 0.75 mm

Using CMR, the TR jet can be visualized based on its signal void with cine imaging. Quantitative TR severity is calculated indirectly. The forward flow volume is obtained from through-plane phase-contrast velocity mapping in the pulmonary artery. After substraction of the forward volume measured in the pulmonary artery from the total RV stroke volume assessed by RV volumetry (ciné steady-state free precession imaging), absolute TR regurgitant volume and fraction can be calculated (59). More recently, 4D-flow CMR has been used for 3D quantification of TR and can correct for through-plane motion as well as eccentricity, with high intra- and interobserver reproducibility and high consistency with 2D phase contrast velocity mapping and echocardiography (60, 61).

### Effective Regurgitant Orifice Area (EROA)

Traditionally, an EROA by proximal isovelocity surface area (PISA) ≥ 40 mm^2^ indicates severe TR. Calculation of the EROA according to the PISA method is based on the assumption of a circular orifice and thus disregards the complexity of the TV, resulting in underestimation of TR severity in one third of patients. Assessment of PISA by 3D-color echocardiography may overcome this limitation by providing a more realistic picture of the actual geometry of the flow convergence zone. However, it may underestimate the actual surface area of the PISA due to angle dependency of the color-Doppler. In addition, PISA only accounts for a single time point and therefore does not integrate the potentially dynamic nature of the flow (42). Alternatively, the EROA can be derived from the quantitative Doppler method, which has been shown to better approximate the planimetric 3D Doppler VC area (14). A possible implementation concept would be the assignment of different cut off values to PISA- and Doppler-derived EROA (52).

### 3D Integrated PISA

**Figure 2:**
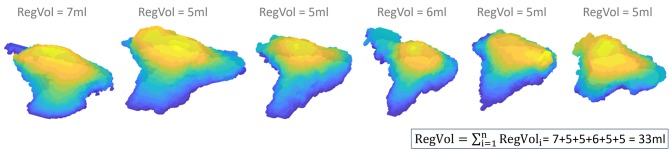
Integrated PISA with three-dimensional reconstruction of 6 PISA_i_ during systole using a Nyquist limit of 22.8 cm/s. This reconstruction highlights the complex shape of the TR PISA and its change in size and shape across systole. The following formula is used to calculate the 3D-PISA_i_ flow: 3D-PISA_i_ flow = 3D-${\text{PISA}}_{\text{i}}^{*}$Nyquist-velocity. The RegVol_i_ of each PISA_i_ is derived using the duration of each frame (1/Volume rate, in this case 0.05 s): RegVol_i_ = 3D-PISA_i_ flow*0.05. The total RegVol is the summation of the RegVol_i_ of each frame.

Instead of using a single PISA to calculate the regurgitant volume, the concept of integrated PISA accounts for temporal changes of the regurgitant flow during systole. With this method, a 3D PISA is reconstructed for each frame of the acquired loop. The flow of each PISA corresponds to its area multiplied by the chosen Nyquist velocity. As explained in Figure 2, the regurgitant volume is obtained by summation according to the duration of each frame. In patients with mitral regurgitation, it best estimates the regurgitant volume compared to CMR with high sensitivity (100%) and specificity (96%) for the detection of severe MR (62).

### Anatomic Regurgitant Orifice Area (AROA)

As recently described for the mitral valve (63), measurements of the anatomic regurgitant orifice by CT are feasible and may be considered as an additional grading tool in patients with discrepant echocardiographic measurements.

**Figure 3:**
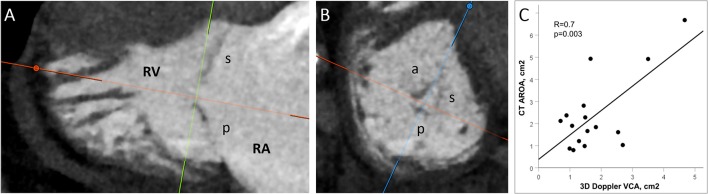
Multiplanar reconstruction of the anatomic regurgitant orifice area (AROA) on MSCT images. **(A)** The reformation planes are adjusted at the leaflet tips during systole (20–40%). **(B)** The regurgitant orifice is delineated on the short axis. The AROA in this case is 0.7 cm^2^. **(C)** Correlation between TEE color-Doppler 3D vena contracta area (VCA) and MSCT AROA obtained with multiplanar reconstruction.

In our experience, the values obtained using multiplanar reconstruction (Figure 3) are generally larger than the corresponding 3D Doppler VC area that rather reflects the effective regurgitant orifice after contraction of the flow stream. However, both parameters significantly correlate (Figure 3C), so that AROA may help to identify patients with severe TR.

### Need for a New Grading Scheme

Since many patients present at a very advanced stage of the disease, current thresholds for severity grading may not sufficiently reflect the variability of the clinical presentation. For this reason, a new grading scheme including the two additional grades “massive” and “torrential” with corresponding cut-offs has recently been proposed (64, 65) and used in clinical studies (66, 67). Preliminary data show an incremental prognostic value of the new classification beyond “severe” (68, 69). In addition, the proposed scheme may allow better appraisal of the results following interventional procedures.

## Assessment of the Right Ventricular Function

RV function has an important prognostic value in patients with TR (33) and, in the absence of elevated afterload, represents a marker of severity and duration of TR mediated volume overload. CMR is considered the gold standard for evaluating the RV dimensions and function due to high spatial resolution and accurate volumetric 3D assessment (without the use of geometrical assumptions) (70).

RV ejection fraction is highly dependent on pre- and afterload, and for this reason, probably suboptimal for the evaluation of RV function in the presence of pulmonary hypertension and/or severe TR (57, 71). Tricuspid annular plane systolic excursion (TAPSE) and tissue-Doppler derived right ventricular excursion velocity (DTI) measured by transthoracic echocardiography are reliable compared to CMR (72). On the other hand, load dependency and variability according to measurement angle represent potential limitations (73). Moreover, the RV contractile pattern shifts after cardiac surgery further decreasing its reliability.

To overcome these specific drawbacks, new methods have been proposed. Using 2D spackle tracking, the longitudinal strain can be derived in all RV segments. This measure correlates well with the RV ejection fraction by CMR (74) and has been validated in patients with various cardiovascular conditions (75, 76) Recent studies have confirmed the high sensitivity of RV strain for the identification of RV dysfunction in the context of severe TR (77, 78). The right ventricular change in pressure over time (dP/dT), as assessed by echocardiography has been proposed as a novel parameter reflecting RV contractility and correlates well with CMR RV ejection fraction (77–79).

In contrast, 3D echocardiographic volumetric quantitation of the RV in different planes is limited by the need for clear delineation of the endocardial borders. Published data, which may overestimate the feasibility of this complex method, show a good correlation for systolic function, but a systematic underestimation of volumes in comparison to CMR (11, 73, 80).

Although less investigated, RV function, dimensions, and volumes may also be reliably obtained from a dedicated 4D electrocardiogram-gated MSCT (81) and normative values have been published (82).

## Patient Selection and Procedural Planning

Multimodality imaging is essential for patient selection as well as procedural planning. Moreover, it may help to anticipate and prevent complications and thereby improve outcomes. Table 3 provides an overview of the specific roles of the different imaging modalities for pre-procedural planning and guiding.

**Table 3:** Overview of the role of the different imaging modalities for preprocedural planning and intraprocedural guiding.

	**Preprocedural**	**Intraprocedural**
Echocardiography/ICE	• TR mechanism and severity • Assessment of RV function • Estimation of RA and pulmonary pressures	• Visualization of catheters and leads • Identification of target points • Assessment of immediate result • Fusion imaging
Multislice computed tomography (MSCT)	• Measurement of annulus and RV dimensions • Asssessment of subvalvular apparatus • Localization of surrounding structures (CS, IVC, SVC, RCA) and implantation site • Implant simulation, 3D printing and access reconstruction	Calculation of optimal fluoroscopic viewing angles
Fluoroscopy	Angiography of RCA	• Navigation of access and in right atrium • Wiring and angiographic depiction of RCA • Valve deployment
Cardiac magnetic resonance	• TR severity • Assessment of RV function	–

### Patient Selection

After thorough assessment of the underlying mechanism of TR, RV function and the exclusion of severe pulmonary hypertension, patients with persisting symptoms despite guideline-directed medical therapy should be evaluated for an intervention by the Heart Team. In patients with concomitant valvulopathy or coronary artery disease requiring surgical revascularization, open-heart surgery remains the first-line treatment. Patients at low surgical risk with isolated severe TR may also be referred for surgical valve repair or replacement, although evidence of an impact on survival is lacking (83, 84) In patients at increased surgical risk, transcatheter techniques may represent a valuable alternative with potential impact on outcomes in terms of heart failure hospitalization and mortality (13).

The surgical experience for valve repair has shown that a tenting area ≥ 1.8 cm^2^, a tenting height ≥ 0.8 cm and a tenting volume ≥ 2 cm3 are predictors of procedural failure for tricuspid repair (85–87). In a similar way, a coaptation depth <10 mm, a central or antero-septal jet location, as well as a coaptation gap of less than about 7 mm have been identified as independent predictors of procedural success for transcatheter interventions (88, 89). In contrast, procedural failure (reduction of TR of less than one grade) and elevated pulmonary pressures were identified as independent predictors of mortality (88). Although no study comparing different devices exist so far, specific system characteristics may better address a given pathology.

The selection of the appropriate transcatheter treatment solution should be based on the severity of annular dilation and jet location. Patients with predominant annular dilation and reasonable leaflet tethering are appropriate candidates for either an annuloplasty device [e.g., Cardioband (67) or TriCinch (90)] or leaflet approximation with either the MitraClip (66, 91, 92) or the Edwards PASCAL system (93). A dedicated system, the Abbott TriClip, is expected to be available soon. For treatment of a central jet, direct annuloplasty may be preferred, while patients with commissural TR are good candidates for leaflet approximation. On the other end of the spectrum, patients presenting late in the course of the disease with advanced RV remodeling, severe leaflet tethering or large coaptation gap should be evaluated for (bi-)caval valve implantation (94–96) or transcatheter TV replacement (79). However, in patients with advanced RV dysfunction, complete elimination of TR through replacement of the valve may precipitate RV failure and eventually lead to cardiogenic shock due to acute afterload mismatch, particularly in the context of preexisting elevated pulmonary vascular resistance and pulmonary hypertension (97). As pulmonary artery resistance may not be reliably reflected by the RV/RA-gradient or the mean invasive pulmonary artery pressure in the presence of TR, it should be calculated based on the values obtained during right heart catheterization. In cases of CIED—lead induced TR, decision should be made individually according to the above mentioned anatomic findings. Data from the TriValve registry showed comparable procedural success and clinical endpoints compared to patients without CIED lead (98).

### Procedural Planning

A comprehensive echocardiographic assessment of the underlying TR mechanism, localization of the regurgitation jet(s) and if applicable, precise CIED-lead location and assessment of its relation to the leaflets (mobile vs. adherent) is crucial for procedural planning of any TV intervention. Especially the TEE short axis view, obtained from transgastric, or the surgical view, acquired by 3D imaging, delivers valuable anatomical information. When aiming for TV repair using leaflet approximation, the exact jet location as well the anticipated implantation strategy (triple orifice vs. bicuspidization), and the number of devices has to be determined. Coronary angiogram should also be part of pre-procedural work-up to confirm patency of the RCA.

Measurement of the TV annulus dimensions is another important step during planning of annuloplasty or valve replacement procedures. In contrast to the left side of the heart, annular dimensions correlate closely with TR severity due to the absence of a fibrous skeleton around the valve, and predominantly functional etiology of TR. A cutoff of ≥ 14–15 cm^2^ for the annular area is indicative of severe TR (14, 99). The complex 3D elliptical shape of the TV annulus is best appraised by TEE or CT using 3D semi-automated imaging techniques that helps to minimize the impact of artifacts due to leads or left heart bioprostheses (11, 99).

According to a recent study, measurement of the tricuspid annulus by CMR is also feasible and reproducible (100).

## Procedural Guiding

Transcatheter tricuspid procedures are guided by 2D and real-time 3D TEE in combination with fluoroscopy, which enables precise positioning of catheters and implants. Near-field views of the TV are obtained using deep transesophageal and transgastric positions of the TEE probe (101). A good acoustic transgastric short axis window is essential to ensure procedural feasibility. The different transcatheter techniques available have variable imaging requirements as detailed in Table 4. The combined skills of the interventional cardiologist and the imaging specialist are essential and equipollent for the success of the procedure. A consistent anatomical nomenclature has been proposed to facilitate intraprocedural communication (102).

**Table 4:** Role of imaging modalities for planning and guiding currently available transcatheter procedures.

	**2D echo**	**3D echo**	**MSCT**	**Fluoroscopy**
Leaflet approximation	+++	++	–	+ (+)
Annuloplasty	+++	+	+++	++
Valve replacement	+++	+++	+++	+
Caval valve implantation	+	–	+++	+++

### Leaflet Approximation

**Figure 4:**
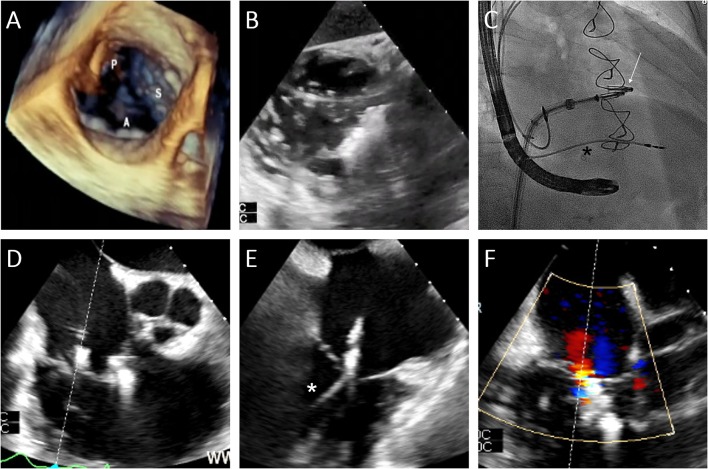
Edge-to-edge repair case. **(A)** Assessment of the baseline valve anatomy using transesophageal 3D echocardiography (A=anterior leaflet; S=septal; p=posterior). **(B)** Orientation of the clip perpendicular to the antero-septal commissure using the transgastric view. **(C)** Insertion of the delivery system into the right atrium under fluoroscopic guidance (projection: RAO 20) after implantation of a MitraClip in the mitral valve (arrow). **(D,E)** Positioning of the clip in the postero-septal commissure using x-plane mid-esophageal view (closer to the aorta is a first clip in the antero-septal commissure, *pacemaker lead). **(F)** Final result after implantation of 2 clips.

Transcatheter leaflet approximation is mainly guided by 2D and 3D TEE (Figure 4A). For the orientation of the implant, a transgastric short axis view (30–50°) of the TV is typically obtained and allows for distinction of the commissures and orientation of the device (Figure 4B). Grasping is performed using an x-plane mid or distal esophageal view (50–75°) cutting either the antero-septal or postero-septal commissure (Figures 4D,E), while the implant orientation is monitored using fluoroscopy (Figure 4C). Bicuspidization or triple orifice technique have been proposed as possible strategies.

#### Transcatheter Annuloplasty

**Figure 5:**
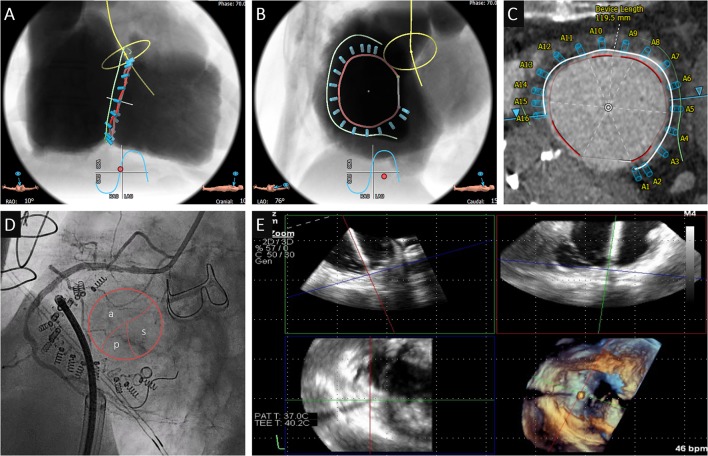
Annuloplasty case. **(A–C)** Preprocedural MSCT planning of the Cardioband implantation (projection A: RAO 10—CRAN 10, B: LAO 76—CAU 15; green line: reconstruction of the RCA). **(A,B)** Anticipated localization of the screws in relationship with the RCA. **(C)** Measurements of the distance between annulus and RCA. **(D)** Angiography of the RCA after Cardioband cinching (projection: LAO 52—CAU 10) with “en face” view of the TV. The ostium and proximal part of the RCA are in close proximity to anterior leaflet while the periphery is close to the posterior leaflet. **(E)** MultiView echocardiography for intra-procedural guiding of screw implantation allowing catheter localization in three planes.

Systematic MSCT analysis plays a crucial role for the planning and guiding of direct annuloplasty. This includes the calculation of optimal fluoroscopic viewing angles (Figures 5A,B), as well as the systematic measurement of the distance between the TV hinge point and the RCA (Figure 5C). Indeed, the RCA is at risk for injury during the procedure, especially if located in close proximity to the site of implantation. An “en face” view of the TV is typically obtained on an LAO fluoroscopic projection and allows for antero-posterior orientation alongside the RCA (Figures 5B,D) and corresponds to the TEE transgastric short axis view (103, 104). In this view, the ostium and proximal part of the RCA surrounds the anterior valve leaflet while the periphery is close to the posterior leaflet. As a further orientation landmark, but also to facilitate a tentative intervention, a coronary guidewire is placed into the RCA during annuloplasty and valve replacement procedures. Visualization of the vessel helps to estimate the distance between the first screws and the aorta that is confirmed by TEE. A two-chamber view with the annulus and RCA in plane is generally obtained with a RAO caudal fluoroscopic projection (Figure 5A) and translates into a 110–130° low-esophageal RV inflow view in TEE (103). The relationship of each screw along the course of the RCA also inform about the position of the catheter in relation to the annulus (more atrial or ventricular). The use of biplane fluoroscopy and 3D echocardiography with multiplanar reconstruction (Figure 5E) enable simultaneous interrogation of several imaging planes.

#### Transcatheter Tricuspid Valve Replacement

Procedural planning of transcatheter tricuspid valve replacement requires detailed anatomic assessment of the tricuspid annulus including measurements of area and perimeter for appropriate valve sizing. Simulation may be used to anticipate access and final valve positioning. Centered position of the valve and deployment are controlled by transesophageal echocardiography (79), and optionally intracardiac echocardiography (105).

#### Caval Valve Implantation

**Figure 6:**
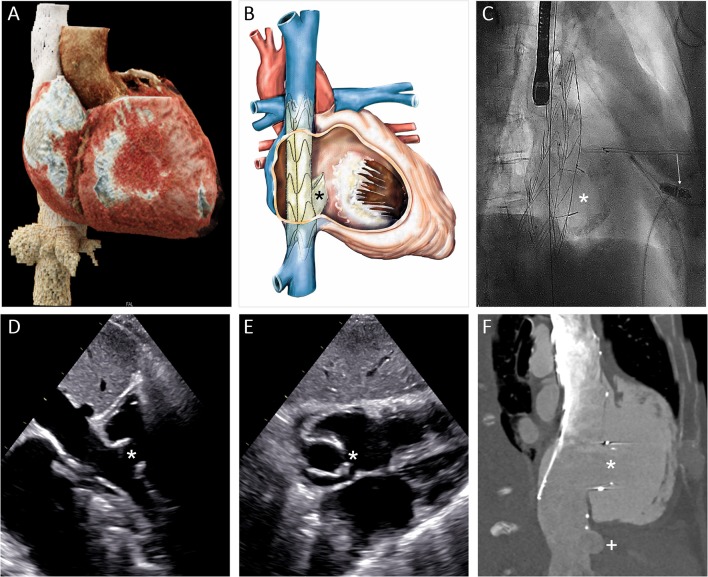
Heterotropic transcatheter caval valve implantation. **(A)** 3D MSCT reconstruction of the vena cava inferior, the liver veins and the right heart cavities. **(B)** Schematic depiction of the NVT Tricento bicaval stenting device. **(C)** Fluoroscopic image of the implanted stent (projection: RAO 45). **(D,E)** Transthoracic echocardiographic imaging of the implanted device in his long and short axis from subxyphoidal at 30-day follow-up. **(F)** Depiction of the prosthesis and its relation to the right atrium and the hepatic vein in computed tomography. (Asterisk: valve element; arrow: leadless pacemaker; plus: hepatic vein).

For heterotopic caval valve placement, MSCT plays a central role to assess the dimensions of the right atrium, identify the ostium of the superior and inferior venae cavae, their angulation and dimensions, as well as the distance to the liver veins (Figures 6A,B). The procedure is then mainly guided by fluoroscopy (Figure 6C), while transthoracic echocardiography and possibly MSCT are used for clinical follow-up (106).

#### Adjunctive Imaging and Visualization Techniques

Intracardiac echocardiography (ICE) is increasingly used to guide transcatheter TV repair, currently as an adjunct to TEE (107–109). Placed in a low right atrial position it enables high resolution imaging of the TV and avoids artifacts from the left side of the heart. Current systems are limited by insufficient far-field imaging quality and the lack of 3D capabilities.

Fusion-imaging integrating echocardiography and/or MSCT, and fluoroscopy require further validation for tricuspid interventions. However, it has the potential to simplify the procedural steps through sophisticated visualization of anatomical structures and catheters/devices in relationship to each other (101, 106, 110).

MSCT provides the necessary information for 3D printing of anatomical models than can be used to simulate and train complex TV procedures.

## Assessment of Result

Assessment of interventional TR treatment efficacy using echocardiography can be challenging, especially after leaflet approximation procedures and/or when multiple TR jets are created. In addition, the implanted devices may produce acoustic shadows impairing correct evaluation of proximal flow convergence and vena contracta. Until now, only *in vitro* studies compared the echocardiographic evaluation of multiple regurgitant orifices with an independent method (111). From a theoretical point of view, only the PISA method (2D or 3D), the volumetric methods and the 3D VCA are appropriate for the quantitative evaluation of multiple regurgitant orifice by summation. Two-dimensional VC widths and jet areas cannot be summed. Changes of the hepatic vein flow patterns are also helpful. However, none of these parameters were tested against an independent method in this setting.

## Conclusion

The tricuspid valve complex challenges imaging specialists and interventional cardiologists in many respects. Patients with TR constitute a heterogeneous and polymorbid population who frequently present late during the course of the disease. Imaging plays a crucial role for the understanding of the natural progression and underlying mechanisms of the disease, as well as for the guiding of transcatheter interventions. Further refinements of current imaging methods will help to better select the appropriate device for the right patient and simplify transcatheter procedures.

## Author Contributions

All authors have made substantial contributions to the conception of the work. It has been drafted by MW and FP and has been critically revised by all authors for important intellectual content. All authors have given their approval for publication of the content and have agreed to be accountable for all aspects of the work in ensuring that questions related to the accuracy or integrity of any part of the work are appropriately investigated and resolved.

## Conflict of Interest

SW reports having received research grants to the institution from Abbott, Amgen, Bayer, BMS, Biotronik, Boston Scientific, CSL Behring, Edwards Lifesciences, Medtronic, Polares and Sinomed. TP reports having received research grants to the institution from Edwards Lifesciences, Boston Scientifc and Biotronik, and speaker fees from Biotronik and Boston Scientific. OK has received speaker's fees from Edwards Lifesciences, and is a consultant for Abbott Structural and Boston Scientific. RH is the Chief Scientific Of ficer for the Echocardiography Core Laboratory at the Cardiovascular Research Foundation for which she receives no direct industry compensation; and has received personal fees from Abbott Vascular, Boston Scientific, Bayliss, Navigate, Philips Healthcare, and Siemens Healthineers. The remaining authors declare that the research was conducted in the absence of any commercial or financial relationships that could be construed as a potential conflict of interest.

## Abbreviations

2D: two-dimensional
3D: three-dimensional
AROA: anatomic regurgitant orifice area
CMR: cardiac magnetic resonance imaging
MSCT: multislice computed tomography
EROA: effective regurgitant orifice area
PISA: proximal isovelocity surface area
RV: right ventricle
TV: tricuspid valve
TEE: transesophageal echocardiography
TR: tricuspid regurgitation
TTE: transthoracic echocardiography
VC: vena contracta
VCA: vena contracta area.
